# Imaging Cells in Flow Cytometer Using Spatial-Temporal Transformation

**DOI:** 10.1038/srep13267

**Published:** 2015-08-18

**Authors:** Yuanyuan Han, Yu-Hwa Lo

**Affiliations:** 1 Department of Electrical and Computer Engineering, University of California, San Diego, California 92093, USA

## Abstract

Flow cytometers measure fluorescence and light scattering and analyze multiple physical characteristics of a large population of single cells as cells flow in a fluid stream through an excitation light beam. Although flow cytometers have massive statistical power due to their single cell resolution and high throughput, they produce no information about cell morphology or spatial resolution offered by microscopy, which is a much wanted feature missing in almost all flow cytometers. In this paper, we invent a method of spatial-temporal transformation to provide flow cytometers with cell imaging capabilities. The method uses mathematical algorithms and a spatial filter as the only hardware needed to give flow cytometers imaging capabilities. Instead of CCDs or any megapixel cameras found in any imaging systems, we obtain high quality image of fast moving cells in a flow cytometer using PMT detectors, thus obtaining high throughput in manners fully compatible with existing cytometers. To prove the concept, we demonstrate cell imaging for cells travelling at a velocity of 0.2 m/s in a microfluidic channel, corresponding to a throughput of approximately 1,000 cells per second.

Cell imaging and high throughput single cell analysis are the primary techniques for studies of cell and molecular biology and medicine^1^,^2^. Microscopy, which is by far the most important imaging tool in biology and medicine, has capabilities to generate cell images of extraordinary details, such as fluorescent images from specific macromolecules, organelles, or subunits of the cells^3^. Yet a microscope yields information via imaging at a relatively low throughput^4^. Given the heterogeneous properties of biological objects such as cancer cells and cells going through different stage of life cycles, much improved understanding of cell and tissue properties can be achieved from the individual properties of a very large population (thousands to millions) of cells. The limited throughput of microscopy techniques has become an impediment for studies of the heterogeneous characteristics of biological samples. On the other hand, flow cytometry is a powerful tool supporting very high throughput analysis, enabling detection of single cell properties at rates from hundreds of cells per second to over 100,000 cells per second^5^,^6^. Flow cytometers can measure and analyze multiple physical parameters of cells, including a cell’s relative size, nuclear granularity, and fluorescence from specific markers or constituents, as each cell in a fluid stream flows through a region of optical interrogation area illuminated by laser beams. However, conventional flow cytometers do not produce the spatial resolution as microscopy does to allow detailed investigation of cell properties that are needed in many applications. As an analogy, flow cytometers can quickly tell male from female over a large group of people without being able to recognize the facial features of each individual, whereas imaging cytometers can reveal the detailed facial features of each person but cannot perform the function fast enough to a large number of people that need to be investigated.

In spite of the above constraint, flow cytometers have been extensively used in biomedical research and playing an increasing role in clinics because of their advantage of high throughput, single cell resolution, and compatibility with cell sorting capabilities^7^,^8^,^9^,^10^,^11^. However, the lack of high spatial resolution that contains valuable phenotypical and morphological information crucial to diagnosis and cell analysis provides a strong incentive to incorporate imaging capabilities into flow cytometry^12^,^13^,^14^. The recently invented parallel microfluidic flow cytometer uses six-pixel one-dimensional spatial information to investigate nuclear translocations, but has very limited spatial resolution to resolve many other sub-cellular compartments and structures compared to a two-dimensional image^15^,^16^. To date the only successful effort in this area is the imaging flow cytometer developed by Amnis/Millipore (e.g. ImageStream)^17^,^18^. Significantly different from all other flow cytometers, the Amnis flow cytometer relies on the time delay and integration (TDI) high-speed charge-coupled device (CCD) camera with a large number of pixels, as opposed to photomultiplier tubes (PMTs) used in almost all today’s flow cytometers to take advantage of PMT’s high speed and superb sensitivity^19^. The Amnis system is much more costly than conventional flow cytometers, and is not ready for integration of cell sorting capabilities due to its unique operation requirements and optics design. As a result, only a very small number (< 5%) of flow cytometers deployed today has acquired the imaging capabilities in spite of the strong desire for such attractive features in flow cytometers^20^,^21^.

In this paper we demonstrate a spatial-to-temporal transformation technique to unify the design of flow cytometer and imaging cytometer. Our design is fundamentally different from the CCD- or CMOS-based technology adopted by nearly all imaging systems today, which requires a relatively long integration time (or exposure time) to capture pictures frame by frame and therefore has speed limitation for imaging cells travelling at high speed^7^,^22^. Rather than using any megapixel imaging devices, we use a specially designed spatial filter placed in front of the PMT detector in the flow cytometer to produce a temporal waveform of the fluorescent or scattering signal. This waveform, encoded by the spatial filter, contains all the information needed to map out the spatial distribution of the signal of a cell, thus allowing construction of the cell image from the temporal waveform. The design is compatible with the existing optic design of conventional flow cytometers, and can be easily implemented to upgrade a conventional flow cytometer to become one with cell imaging capabilities at minimum cost. To prove the concept, we show single cell images of A549 human lung adenocarcinoma epithelial cells in a flow cytometer prototype shown in Fig. 1a. The flow speed of the cells is 0.2 m/s, corresponding to a throughput of approximately 1,000 cells per second.

## Results

### Experimental setup of the imaging flow cytometer

**Figure 1:**
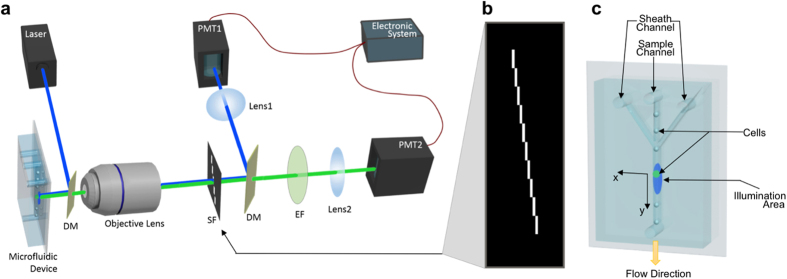
Implementation of imaging flow cytometer. (**a**) Schematic diagram of the imaging flow cytometer system. DM, dichroic mirror; SF, spatial filter; EF, emission filter; PMT, photomultiplier tube. (**b**) Spatial filter design that has ten 100 um by 1 mm slits positioned apart in the way of one is immediately after another in both x-direction and y-direction [labeled in (**c**)]. (**c**) Microfluidic device in which suspended cells are controlled by sheath flow to travel in the center of the microfluidic channel at a uniform velocity.

A diagram of the imaging flow cytometer prototype is shown in Fig. 1a. The system consists of three main units: 1) fluidic system for introducing cells into a microfluidic channel, 2) optical system for illumination and detection of the light signals, 3) electronic system for data acquisition and processing. Except for the spatial filter detailed in Fig. 1b, all other parts are common to any conventional, generic flow cytometers, so the results obtained here can be generalized to other flow cytometers with only minor modifications.

At first the suspended cells are introduced into the microfluidic channel and hydrodynamically focused by sheath flow, ensuring that the cells travel in the center of the fluidic channel at a uniform velocity^23^. The fluorescence emission and backscattering light from the sample are detected by two individual PMTs in a wide-field fluorescence microscope configuration. Here cells are flown in a microfluidic channel made of soft-molded PDMS bonded to a glass substrate. To accommodate the geometry of the microfluidic device, the laser beam is introduced to the optical interrogation site in the fluidic channel by a miniature 45-degree dichroic mirror positioned in front of a 50X objective lens (NA = 0.55, working distance = 13 mm). The size of the 45-degree dichroic mirror is small enough to allow the backscattering light (147° to 168° with respect to the normal incident light) to pass the dichroic mirror and enter the objective lens. A spatial filter having the pattern shown in Fig. 1b is inserted in the detection path right at the image plane of the optic system. Both the fluorescent and backscattering light from a travelling cell are collected by the objective lens and pass the filter before reaching their respective PMT detectors. Another dichroic mirror splits the light by its spectrum to route the desired emission bands to the appropriate PMTs. Finally, the output of each PMT is sent to a computer and processed to generate cell images from fluorescence and back scattering. Although the system in Fig. 1 shows only one PMT for detection of fluorescent signal, it is straightforward to add more PMTs and, if necessary, more excitation laser beams, to produce multi-color fluorescent signals as in any conventional flow cytometers.

### Restoring cell images from light intensity profiles

The concept of spatial-to-temporal transformation can be mathematically formulated in the following:


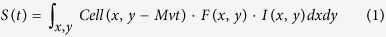


where *S*(*t*) is the measured PMT signal, *Cell* is the two-dimensional cell fluorescence or scattering intensity profile, *F*(*x, y*) is the characteristic function of the spatial filter, *I*(*x, y*) is the intensity profile of laser illumination, *y* is along the cell travelling direction and x is along the transverse direction, and *M* is the magnification factor of the optical system pertaining to the flow cytometer. As the cell travels in the microfluidic channel at a speed *v*, the image projected onto the spatial filter travels at an effective speed of *Mv*. To simplify the mathematical process of solving for *Cell* in Equation (1), here we choose *F*(*x, y*) to be a series of rectangle function represented in equation (3) and *I*(*x, y*) to be a constant from a laser beam of uniform intensity.


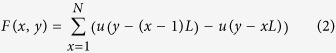


where *x *= 1,2…,*N* is the number of row in the spatial filter, *L* is the length of the rectangular slit that transmits fluorescent or scattering light. We can rewrite Equation (1) as


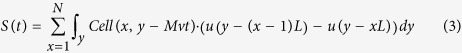


We can solve for “*Cell*” by taking the time derivative of Equation (3)


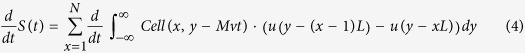


Assuming the cell orientation does not change within such a short time interval, one can represent Equation (4) as the following:


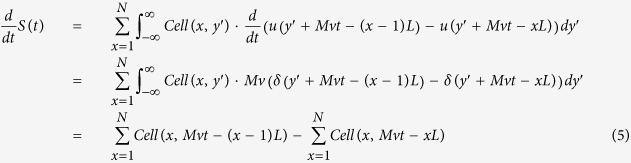


To obtain Equation 5, we have used *y*′ = *y*−*Mvt*. When the cell size does not exceed the slit length *L*, within the specific time interval 
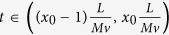
, 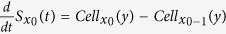. As a result, the cell image can be constructed from the following relation:


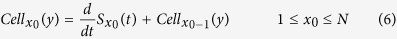


Generally, with the spatial filter described above inserted at the image plane, fluorescence from different parts of the cell will pass different slits at different times. As a result, the waveform of the fluorescent signal from the PMT consists of a sequence of patterns separated in time domain, and each section of the signal in the time domain corresponds to the fluorescent signal generated by each particular segment of the cell. After the light intensity profile over each slit is received, the cell image of the entire cell can be constructed by splicing all the profile together. In our prototype, the spatial filter contains ten 100 μm by 1 mm rectangular slits positioned in sequence as shown in Fig. 1b. An alternative spatial filter that has two slits of 100 μm by 1 mm in x- and y-direction, respectively, in front of the slit tandem can be applied for precise calculation of cell traveling speed for each cell. With a 50X objective lens (M = 50) in our prototype, the filter design allows us to construct the fluorescent or scattering image of a travelling cell no larger than 20 μm by 20 μm using the algorithm described in Equation (6), which requires a minimum amount of computations and is suitable for high-throughput, real-time image-based cell classification and sorting.

**Figure 2:**
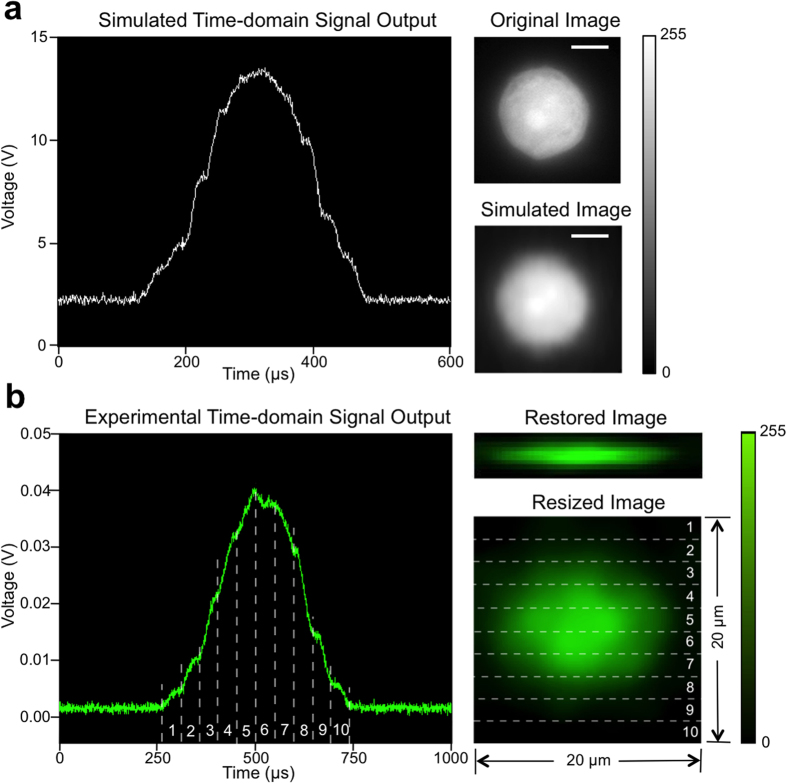
Illustration of restoring cell images from PMT signals. (**a**) Simulation result: result of time-domain light intensity signal from simulation, original cell image used as input for simulation, and corresponding restored image. Scale bar is 5 μm. (**b**) Experimental result: time-domain PMT output signal of fluorescent light from a A549 cell stained with CellTrace CFSE, corresponding original fluorescence image restored by algorithm, and corresponding resized fluorescence image to show the real size of the cell. The numbered regions segmented by dashed lines demonstrate the correspondence between the time-domain signal and the resulting image. Size is labeled in figure.

To demonstrate the feasibility of this approach by simulation, we use a cell image as the cell under test and have the cell travel through an illumination beam spot at a speed of 0.24 m/s. The fluorescence light that passes the slits on the spatial filter is sampled at a rate of 500 kHz. Figure 2a shows the original cell image, the time-domain output signal through the spatial filter, and the restored cell image using the aforementioned algorithm. Compared to the original cell image, the restored image from simulation shows identical features.

During every experiment, once the cells are injected into the microfluidic channel, a two-slit filter is inserted at the image plane to determine the cell travelling velocity, followed by applying the aforementioned spatial filter. The time-domain signal for each cell is captured by thresholding the PMT readout. Figure 2b shows the experimental result of a typical PMT signal and the fluorescence cell image constructed from the PMT signal using equation (6). The spatial resolution of the restored image in x- (transverse) direction depends on the number of the slits on the spatial filter, and in y- (cell travelling) direction depends on the sampling rate and cell flow rate. In the original image restored by the imaging flow cytometer (shown in Fig. 2b), the effective pixel size is 2 μm in x-direction and about 0.4 μm in y-direction. The recovered image is then resized to 80 pixel by 80 pixel to better represents a 20 μm by 20 μm area in the object plane in the microfluidic channel. Using a 50X/0.55NA objective lens, 500 kHz sampling rate for acquiring PMT signal, and 0.2 m/s cell travelling speed that is given by 12 μL/min sample flow rate and 120 μL/min sheath flow rate, the effective size of the pixel in y-direction is 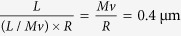 which is smaller than the Rayleigh Criterion, thus resulting in a diffraction-limited resolution in y-direction. Here R is the sampling rate of PMT readout in this calculation.

**Figure 3:**
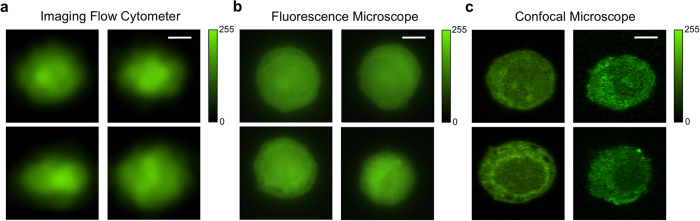
Comparison of spatial filter based flow cytometry imaging and wide-field fluorescent imaging. All images are of A549 human lung adenocarcinoma epithelial cells, stained with CellTrace CFSE. (**a**) Representative imaging flow cytometer reconstructed fluorescence images of cells flowing at a velocity of 0.2 m/s. Objective lens: 50X/0.55 (**b**) Representative wide-field fluorescence images of stationary A549 cells taken by fluorescence microscope. Objective lens: 100X/0.90. (**c**) Representative confocal microscope images of stationary A549 cells. Objective lens: 63X/1.30. Size of all image crops is 20 μm by 20 μm; scale bar is 5 μm.

The imaging flow cytometer system enables fast fluorescence imaging using only a single PMT instead of pixelated CCD. Figure 3a shows representative fluorescence images of fluorescently labeled A549 cells flown at 0.2 m/s in a microfluidic channel, producing a throughput of around 1,000 cells/s. For comparison, Fig. 3b shows images of the stationary fluorescently labeled A549 cells between a glass slide and a coverslip captured by a fluorescent microscope with a CCD camera under at least 50 ms exposure time; Figure 3c shows images from same batch by a confocal microscope. The resulting images from the imaging flow cytometer appear to be similar to the images of still cells from a fluorescence microscope, even though in the imaging flow cytometer, the cells are travelling at a speed of 0.2 m/s and the signals that give rise to the cell images are detected by a single PMT in a setup and configuration compatible with conventional flow cytometers. Table 1 shows comparisons in size and shape descriptors of cell images acquired by our imaging flow cytometer prototype and fluorescence microscope. Based on 100 cell fluorescence images randomly picked from each group, the measured cell size, circularity, and solidity of the images restored by the imaging flow cytometer are highly consistent with the images taken by the fluorescence microscope, with exception of the aspect ratio defined as the ratio of the major axis to the minor axis of the fitted ellipse. The appreciable difference in the cell aspect ratio between the imaging flow cytometer and fluorescent microscopy is attributed to cell deformation by the fluidic dynamic shear stress, carrying information about cell stiffness, a property of biological significance.

### Backscattering image

Our spatial-temporal transformation technique is not restricted to specific modes of signals. In the following we show that the approach is capable of combining fluorescence images with backscattering images. The backscattering images captured by our imaging flow cytometer system reveal the unique properties of cell nuclei as effective markers for applications such as disease diagnosis, cell classification, and cell cycle monitoring^24^. Cellular components with higher concentration of macromolecules exhibit a higher refractive index^25^,^26^ than the background. These refractive index variations will scatter light when the cell is illuminated by visible light. The size and refractive index distributions of the scattering regions determine the angular distributions of the scattered light^27^,^28^. The majority of human cancers originate in the epithelial cells, so the backscattering imagery can potentially benefit the diagnosis of early cancer and intra-epithelial neoplastic changes. To demonstrate the feasibility of the backscattering imaging function for cell nucleus monitoring, A549 cells that are going through different life cycles are tested using the imaging flow cytometer.

To observe cells in different stages, A549 cells are cultured with inhibitors to stop their growth at different development stages^29^. Mitomycin is used to stop the cell growth at G1 phase where the biosynthetic activities of cells are activated to form necessary proteins for the next phase (S phase). Separately, nocodazole is used to arrest the A549 cells at G2/M phase, more specifically at the prometaphase. Being arrested at the prometaphase, the nuclear membrane breaks down and the constituents of nucleus are distributed within the cytoplasm. Lacking a well-defined nucleus confined by the nucleus membrane, the cell has generally stronger but no well-defined contour in light scattering.

**Figure 4:**
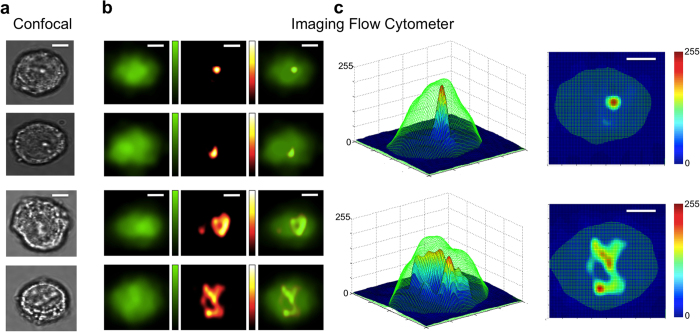
Backscattering and fluorescent cell images from spatial filter based imaging flow cytometry. All images are of A549 human lung adenocarcinoma epithelial cells, stained with CellTrace CFSE, flowing at a velocity of 0.2 m/s. (**a**) Representative confocal images of G1 (top two) and G2/M (bottom two) arrested cells. (**b**) Representative imaging flow cytometer images of G1 (top two rows) and G2/M (bottom two rows) arrested cells. Fluorescence images are shown in left column, backscattering images are shown in middle column, and overlay images are shown in right column. Green colorbar representing intensity from 0 to 255 is applied for fluorescence images. Hot colorbar representing intensity from 0 to 255 is applied for backscattering images. (**c**) 3-dimensional plots for overlay of fluorescence (green mesh) and backscattering (jet surface) images of G1 (top) and G2/M (bottom) arrested cells. Jet colorbar representing intensity from 0 to 255 is shown. Size of all image crops is 20 μm by 20 μm; all scale bars are 5 μm.

Figure 4a shows representative confocal images of stationary cells arrested at G1 phase and prometaphase. Figure 4b shows the fluorescence images, backscattering images, and superposition of these two images of travelling cells (0.2 m/s) in our imaging flow cytometer as shown in Fig. 1. Images acquired by both systems show the general characteristics that cells arrested at G1 phase possess a clearly defined scattering center from the nucleus. In contrast, cells at prometaphase show overall stronger scattering intensities because of higher concentration of nucleic acids and proteins but no well-defined scattering center due to lack of nuclear membrane.

To embody the volume of the scattering cellular components within the cells that are arrested at specific phases, 3-dimensional contour plots for backscattering images overlaid on the fluorescence images are shown in Fig. 4c. Again both the backscattering images from the method of spatial-temporal transformation and the confocal images show consistent subcellular features: cells at G1 phase have a condensed scattering center; and cells at prometaphase have a more distributed scattering region.

**Figure 5:**
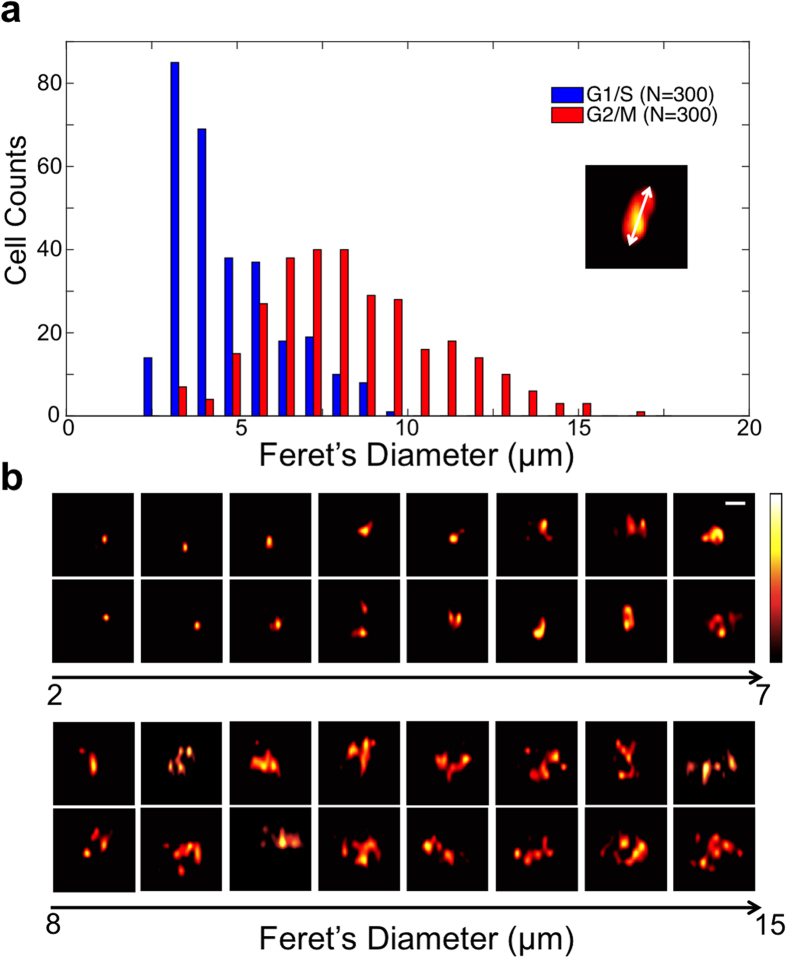
Differences of the backscattering images of cell arrested at G1 phase and G2/M phase. All images are of A549 cells flowing at a velocity of 0.2 m/s. (**a**) Histogram of the Feret’s diameters of the G1 (blue bar) and G2/M (red bar) arrested cells’ backscattering images. 300 cell images from each group are measured. The sketch shows the definition of the Feret’s diameter: the longest distance between any two points along the object’s boundary, also known as maximum caliper. (**b**) Two example backscattering images for each bin in the histogram. Hot colorbar represents intensity from 0 to 255. Sizes of all cell backscattering images are 20 μm by 20 μm; the scale bars is 5 μm.

For traditional flow cytometry, a histogram is one common way to provide information about a cell population or subpopulation. The parameter associated with the histogram is nothing more than fluorescence or scattering intensity. It is more informative if image of every single cell under test is available for flow cytometry tests, so that making decisions about gating can be no longer blind to the sample attributes. Moreover, not only the light intensity can be quantified, but also many morphological measurements can be performed on account of the available images of the cells under tests. To compare the backscattering images of cells arrested at G1 and G2/M phase, Fig. 5a shows the histogram of the Feret’s diameter, also known as maximum caliper. The G1 cells, shown in blue bars, tend to have Feret’s diameter of 3 to 4 μm, and G2/M cells, shown in red bars, have larger Feret’s diameter. Instead of pure numerical expression, Fig. 5b shows two example backscattering images for each bin from 2 μm to 15 μm in the histogram.

## Discussion

We have demonstrated a spatial-temporal transformation technique that enables traditional flow cytometers to capture fluorescence and backscattering images of cells travelling at high speed in fluid stream. The image quality is comparable to the conventional fluorescence microscopy imaging for still cells using a CCD or CMOS camera. The spatially distributed backscattering plots generated by our system reveal not only the commonality of cells of the same type but also the inhomogeneity of them, exemplified by the same cell type undergoing different life cycles. Because of the simplicity of the design and the use of PMTs rather than CCDs for construction of cell image, our approach can convert or retrofit existing flow cytometers into systems with single cell imaging capabilities. While in our proof-of-concept demonstration, we show imaging results for two parameters (i.e. one-color fluorescence and backscattering), our method can be applied to produce cell images of multiple fluorescent colors with additional dichroic mirrors and PMTs. Furthermore, our method can work at higher fluid flow rate for higher spatial resolution and throughput with high-speed digital data acquisition electronics.

## Methods

### Microfluidic device fabrication

The microfluidic device was fabricated using the standard polydimethylsiloxane (PDMS) replica molding methods. The Si mold masters were fabricated by the reactive ion etching (RIE) process. The microfluidic channels were drawn in AutoCAD (Autodesk, Inc.), and were photolithographically defined using negative photoresist (NR9-1500PY, Futurrex, Inc.), which serves as an etch mask during the following dry-etching process. A 4-inch silicon wafer was etched at room temperature using inductively coupled plasma (ICP) reactive ion etching (ICP RIE; Plasmalab 100, Oxford Instruments) to reach a depth of 75 μm. Plasma ignited from a mixture of O_2_ and SF_6_ gases performed the etching and sidewall passivation, resulting in smooth and vertical channel walls. The Si mold master was silanized by vapor deposition of trichlorosilane (TCI Inc.) to facilitate PDMS de-molding. A replica was made by casting the PDMS (Sylgard 184, Dow Corning), mixed in the standard 10:1 ratio of base to curing agent, over the Si mold master. After thermal curing in the oven for 3 hours at 65 °C, the PDMS layer was peeled off of the mold, and holes for inlets and outlets were punched. The surfaces of the demolded PDMS layer and a glass wafer were both treated with UV/Ozone to facilitate covalent bonding of them to form microfluidic channels for the imaging flow cytometer experiment.

### Optical System

A 25 mW 488-nm single-mode fiber coupled laser (FTEC2, Blue Sky Research) has a circular beam shape with Gaussian energy distribution. A top-hat beam shaper (Osela, Inc.) is used to convert the Gaussian beam to a uniform top-hat profile, which illuminates an area of 100 μm (x-direction) by 350 μm (y-direction). The fluorescence passing the miniature dichroic mirror with 500 nm cutoff wavelength (ThorLabs) and the scattering light are collected through a 50X, 0.55NA objective lens (Mituyoyo). The light intensity signal in each channel is acquired by a PMT (H9307-02, Hamamatsu) and recorded using LabVIEW. The saved raw data are processed in MATLAB implementing the aforementioned algorithm.

### Spatial filter fabrication

The design of spatial filter was drawn in AutoCAD and printed to a transparency mask at 20,000 dots per inch (dpi). A layer of negative photoresist (NR9-1500PY, Futurrex, Inc.) was spun at 3,000 rotations per minute (rpm) on a 6-inch glass wafer. The wafer was heated on a hot plate at 150 °C for 3 minutes then exposed to UV light (EVG620NT, EV Group) through the transparency mask. Post UV exposure, the wafer was baked at 100 °C for another 3 minutes before development in RD6 (Futurrex, Inc.) for 12 seconds. A film of 200 nm thick aluminum was sputtered onto the glass wafer. After metal lift-off, the patterns of the spatial filter were formed and the glass wafer was diced into 15 mm by 15 mm pieces. To help hold the spatial filter in the flow cytometer system, the spatial filter having ten 1 mm by 100 μm slits was mounted to a sample holder fabricated by 3D printing method.

### Preparation of cell samples

The A549 human lung adenocarcinoma epithelial cell samples were harvested from culture and labeled with CellTrace CFSE Cell Proliferation Kit (Life technologies) that has excitation and emission peaks at approximately 492 nm and 517 nm, respectively. After incubation in 4% formaldehyde for 20 min, the A549 cells were washed and resuspended in phosphate buffered saline (PBS). Before every imaging experiment, the suspension was diluted in PBS to a concentration of 200 cells/μl. To arrest A549 cells at the G1 phase, Mitomycin (10 μg/ml) dissolved in DMEM, mixed with 0.5% FBS and 1% PS, was added to the culture medium and then the cells were incubated for 3 hours prior to the experiment. To arrest cells at the G2/M phase, 50 ng/ml of nocodazole in DMEM, mixed with 0.5% FBS and 1% PS, was added to the culture medium and the cells were cultured for 16 hours. Cells arrested at the designed phase were washed with PBS and suspended in 4% formaldehyde. After keeping the cell suspension at room temperature for 20 minutes, the sample was centrifuged at 1000 rpm for 10 min and the supernatant of cell suspension was carefully discarded. After washing the sample left in the tube with PBS, the fixed cells were resuspended in PBS to the concentration of 200 cells/μL.

### Measurements of Cell Morphological Features

The output images from the imaging flow cytometer represent a 20 μm by 20 μm area; the cell fluorescence images from the fluorescence microscope (BZ-9000, Keyence) are also cropped to the same area. To measure the morphological features of both the cell images restored by the imaging flow cytometer and taken by the fluorescence microscope, all images are processed using ImageJ. Two image sequences that include 100 images from each system are imported to ImageJ. After setting the measurements to include area and shape descriptors, the command “Analyze Particles” is used to measure the concurrently thresholded images. The mean values and the standard deviations in Table 1 are calculated based on the results from ImageJ. For the Feret’s Diameter measurements of backscattering images, 300 images from each of the G1 arrested cells and the G2/M arrested cells are analyzed using the same method in ImageJ. To avoid multiple measurements in one image due to the disconnected patterns, especially in the backscattering images of G2/M arrested cells, all the images, including both G1 and G2/M cell images, are smoothed twice and concurrently thresholded, and only the largest Feret’s Diameter in every image is recorded.

## Additional Information

**How to cite this article**: Han, Y. and Lo, Y.-H. Imaging Cells in Flow Cytometer Using Spatial-Temporal Transformation. *Sci. Rep.* **5**, 13267; doi: 10.1038/srep13267 (2015).

## Figures and Tables

**Table 1:** Comparison of cell morphological features between fluorescence images restored by our imaging flow cytometer prototype and taken by fluorescence microscope based on 100 cell images from each group.

Cell Images from	Area (um^2)		Circularity		Solidity		Aspect Ratio	
	Mean	St. D.	Mean	St. D.	Mean	St. D.	Mean	St. D.
Imaging Flow Cytometer	126.77	3.199	0.82	0.028	0.96	0.007	1.52	0.088
Fluorescence Microscope	128.74	4.511	0.87	0.048	0.96	0.013	1.08	0.064

Size is the above-background area of the cell image; circularity equals to 4πA/P^2^, where A is area, P is perimeter of the cell image; solidity is the ratio of area to convex area; aspect ratio is the ratio of major axis to minor axis of the cell image’s fitted ellipse.
